# Pneumatic Tourniquets

**Published:** 1904-04-16

**Authors:** 


### PNEUMATIC TOURNIQUETS.

It is to be doubted whether the fact is sufficiently appreciated that the clumsy use of a tourniquet may result in serious damage to the soft parts that are compressed by it. It frequently happens that in order to prevent hæmorrhage during operations on the upper extremity a piece of stout indiarubber tubing is tightly twisted round the arm. In the relaxed condition of the muscles under anæsthesia, and especially where the muscles are wasted from disease, there is a danger of so compressing the tissues between the tourniquet and the bone as to cause serious damage. Thus every degree of nerve-injury from temporary paresis to permanent paralysis may result, though, of course, the latter is very exceptional and requires much force for its production. Dr. H. Cushing [1] records that he has observed during the last two years no fewer than eight cases of pressure paralysis caused by the use of tourniquets. In the majority of these cases the paralysis followed the application of a tourniquet to the arm. Another drawback to the use of an elastic band when tightly applied for the arrest of hæmorrhage is the ensuing vaso-motor palsy. In an amputation this produces the wet stump so annoying to the operator, as it necessitates drainage, which otherwise might have been avoided. To surmount these objections, Dr. Cushing suggests the use of an inflatable tourniquet. He has made use of such a device and finds it most efficient. He uses indiarubber tubing thicker than that usually employed, measuring 3 cm. on the flat, and made of less distensible rubber and of such quality that it will stand boiling. This is fastened in the required place, and then inflated with a bicycle pump until the circulation is arrested, as observed by palpating a peripheral artery. In this way the circulation may readily be controlled without the use of undue and possibly harmful pressure on the soft parts. An additional advantage is that the pressure can be removed and reapplied, as is often necessary in the course of an amputation, for example, without any troublesome manipulations and disturbing of the field of operation. This apparatus is not only of service in operations upon the extremities, Dr. Cushing finds, but is admirably adapted for use in operations upon the scalp and cranium, since it can be used to control the bleeding without being followed by pressure neuritis of the supra-orbital and sub-occipital nerves, as sometimes occurs after the ordinary indiarubber tubing has been used. By fixing the tubing round the forehead and sub-occipital region, the blood supply of nearly the whole scalp may be kept under control. This is a matter of considerable advantage, as no time need be lost, in a trephining operation for example, in securing the cut vessels, accurate suturing of the skin incision, and firm bandaging being amply sufficient to control hæmorrhage after the tourniquet has been removed.

[1] Medical News, March 26.

